# Prolonged feeding of terrestrial isopod (*Porcellio scaber*, Isopoda, Crustacea) on TiO _2_ nanoparicles. Absence of toxic effect

**DOI:** 10.3897/zookeys.176.2463

**Published:** 2012-03-20

**Authors:** Sara Novak, Damjana Drobne, Anja Menard

**Affiliations:** 1 University of Ljubljana, Biotechnical Faculty, Department of Biology, Vecna pot 111, Ljubljana, Slovenia; 2 Centre of Excellence in Advanced Materials and Technologies for the Future (CO NAMASTE), Jamova 39 Ljubljana, Slovenia; 3 Centre of Excellence in Nanoscience and Nanotechnology (CO Nanocenter), Jamova 39, Ljubljana, Slovenia; 4 University of Ljubljana, Faculty of Pharmacy, Askerceva cesta 7, Ljubljana, Slovenia

**Keywords:** Isopods, *Porcellio scaber*, TiO_2_ nanoparticles, prolonged feeding, toxic effects

## Abstract

Nanoparticles of titanium dioxide are one of most widely used nanomaterials in different products in everyday use and in industry, but very little is known about their effects on non- target cells and tissues. Terrestrial isopods were exposed to food dosed with nano-TiO_2_ to give final nominal concentration 1000 and 2000 µg TiO_2_/g dry weight of food. The effects of ingested nano-TiO_2_ on the model invertebrate *Porcellio scaber* (Isopoda, Crustacea) after short-term (3 and 7 days) and prolonged (14 and 28 days) dietary exposure was assessed by conventional toxicity measures such as feeding rate, weight change and mortality. Cell membrane destabilization was also investigated. No severe toxicity effects were observed after 3, 7, 14 or 28 days of dietary exposure to nano-TiO_2_, but some animals, particularly those exposed to lower concentrations of nanoparticles, had severely destabilized digestive cell membranes. It was concluded that strong destabilization of the cell membrane was sporadic, and neither concentration- nor time-related. Further research is needed to confirm this sporadic toxic effect of nanoparticles.

## Introduction

During the last decade the presence of nanomaterials has increased extraordinarily, and information on their toxicity is urgently needed. Nanomaterials have unique physical and chemical properties as a result of their small particle size, shape, conductivity and surface characteristics. TiO_2_ nanoparticles are most commonly encountered nanoparticles and as a consequence they could become a substantial environmental pollutant. Nanoparticles of TiO_2_ have been shown to have different types of effects *in vivo* (Menard et al. 2011), although their toxic potential appears not to be very pronounced. Many studies indicate that the effects of nanoparticles differ significantly from those of soluble pollutants. There are some indications that nanoparticles may have some nanoparticle-specific effects on biological systems.

Many reports using terrestrial isopods as toxicity test organisms for chemicals and particles in laboratory single-species tests can be found in the literature, and *Porcellio scaber* (Isopoda, Crustacea) is among themost frequently used species in such studies. The species was found to be suitable in tests of the effects of elevated concentrations of metals (Drobne and Hopkin 1994, 1995, Jereb et al. 2003, Zidar et al. 2005), biocides (Staak et al. 1998, Stanek et al. 2006), veterinary drugs (Kolar et al. 2010, Zizek et al. 2011), and nanoparticles (Drobne et al. 2008, Jemec et al. 2008, Pipan-Tkalec et al. 2010, 2011). In these tests, several endpoints have been assessed, including biochemical biomarkers, histopathological changes, behavioral response and physiological measures and organism level responses. The selected biomarkers vary in their sensitivity.

Conventional measures of toxicity such as investigations of growth, reproduction, and life-cycle are not the most suitable when terrestrial isopods are the test organism. Rates of growth in terrestrial isopods over several weeks are variable even for a single individual (Van Capelleveen 1987); reproduction is difficult to assess because after mating, females may retain the sperm for a long period before reproducing, and the life cycle of most terrestrial isopods is relatively long, often more than 6 to 8 months (Drobne 1997).

In toxicity tests with isopods however, feeding parameters have proved to be an integrated organism-level response, appropriate evidence of the effects of chemicals. Feeding rate changes are relatively fast and have been observed in relation to added metals or organic chemicals. Reduced feeding rate in comparison to controls was recorded after exposure of isopods to metals and biocides (Drobne and Hopkin 1995, Drobne at al 2008) and in addition, measurements of feeding rate are non-invasive and feeding rates can be recorded both during and after the exposure (Drobne 1997) Finally, after exposure, many additional biomarkers at lower levels of biological complexity can be analyzed too.

Recently, studies on the effects of nanoparticles were performed with *Porcellio scaber*. When added to food, TiO_2_ particles had no adverse effect on the feeding rate of *Porcellio scaber* after 3 or 14 days dietary exposure (Drobne et al. 2008) to up to 1000 μg/g dry food. In this study TiO_2_ nanoparticles (nano-TiO_2_) were reported even to enhance the feeding rate of *Porcellio scaber*. Similarly, Jemec et al. (2008) reported no reduction in food consumption by *Porcellio scaber* when feeding on nano-TiO_2_ (3000 μg/g dry food) for 3 days. In a study by Pipan-Tkalec et al. (2010), in which animals were exposed for 4 weeks to food dosed with 2000 or 5000 μg ZnO nanoparticles/g dry weight of food, the feeding rate was not affected by the elevated concentrations of Zn in the food and no adverse effect on feeding behavior was recorded after 14 days of exposure to silver nanoparticles up to 5000 µg nano-Ag/g dry weight of food (Pipan-Tkalec et al. 2011). These studies indicate that while feeding parameters are not affected within one or two weeks of exposure, they will be affected ultimately along with biomarkers at lower levels of biological complexity.

In the present study, the effects of nano-TiO_2_ on the model invertebrate *Porcellio scaber* (Isopoda, Crustacea) after brief (3 and 7 days) and prolonged (14 and 28 days) dietary exposure are examined. We discuss the toxic effect of ingested TiO_2_ nanoparticles on this terrestrial isopod. The feeding rate was used as evidence of a toxic effect and the cell membrane destabilization as a measure of a cytotoxic effect. We have found that after 14 days of exposure to nano-TiO_2_, the feeding rate of *Porcellio scaber* was not significaly affected, but cell membranes were destabilized in more than 40% of the population. If cell membrane destabilization leads to cytotoxicity, prolonged exposure of *Porcellio scaber* to nano-TiO_2_ will result in toxic effects which can be assessed by conventional toxicity measures. If there is a reduced feeding rate after prolonged exposure, this will confirm the time- and dose-dependency of the effects of nano-TiO_2_ which has been seen with other materials.

## Materials and methods

### Chemicals

Acridine orange (AO), ethidium bromide (EB) and titanium dioxide nanoparticles (nano-TiO_2_) were purchased from Sigma-Aldrich. The nano-TiO_2_ was the same as was used in our earlier experiments (Valant et al. 2009) and was supplied as a powder, guaranteed 99.7% pure, with an anatase crystal structure, average particle size <25 nm and surface area between 200 and 220 m^2^/g.

### Model organisms

Terrestrial isopods (*Porcellio scaber*, Isopoda, Crustacea) were collected in July and August 2010 at location (46°4'20"N, 14°26'51"E) near Ljubljana, Slovenia. The animals were kept in a terrarium filled with a layer of moistened soil and a thick layer of partly decomposed hazelnut tree leaves (*Corylus avellana*), at a temperature of 20 ± 2°C and a 16:8-h light:dark photoperiod. Adult animals of both sexes, weighing more than 30 mg, were used in the experiments. If moulting or the presence of marsupia were observed at any time, the animals were removed from the experiment in order to keep the investigated population as physiologically homogenous as possible.

### Characterization of nanoparticles

Nanoparticles were inspected with transmission electron microscopy (TEM), Brunauer-Emmett-Teller (BET) analysis, dynamic light scattering (DLS) and X-ray powder diffraction techniques. TEM micrographs were published in a previous report by Valant et al. (2009). Before and after the exposure of isopods to nano-TiO_2_, three randomly selected pieces of leaves were dried, attached to mounts with silver paint (SPI), gold/palladium-sputtered (Sputter Coater SCD 050, BAL-TEC, Germany) and investigated at the Institute of Metals and Technology, Ljubljana, Slovenia with a field emission scanning electron microscope (FE-SEM; Jeol JSM-6500F). Energy dispersive X-ray analysis (EDX; EDS/WDS Oxford Instruments INCA, Jeol JSM-6500F) was used to confirm the chemical composition of nanoparticles on the leaves.

In the DLS analysis, the dispersions of nanoparticles (100 µg nano-TiO_2_/ml distilled water) were examined with a 3D DLS-SLS spectrometer (LS Instruments) which allows the assessment of the hydrodynamic radii of particles in extremely turbid suspensions by a 3D cross-correlation technique that eliminates light scattering. The light source used was a HeNe laser operating at a wavelength of 632.8 nm and scattering was measured at an angle of 90°. At higher concentrations of nanoparticles (1000, 2000 µg nano-TiO_2_/ml distilled water), measurements were not possible, due to the low transparency of the samples (Valant et al. 2009). The same particles were also tested in some other studies which provided a more detailed description of their characteristics (Valant et al. 2009).

### Experimental design

Hazelnut leaves were dried at room temperature, and cut into pieces weighing ~100 mg. The TiO_2_ nanoparticles were suspended in distilled water to obtain different final concentrations (1000 and 2000 µg/ml). In a control group, the leaves were treated with pure distilled water. A suspension of particles was brushed onto the abaxial leaf surface and the leaf was allowed to dry, giving final nominal concentrations of nanoparticles on the leaves of 1000 and 2000 µg nano-TiO_2_ per gram (dry wt) of leaf.

A single hazelnut leaf treated with either distilled water or nano-TiO_2_ suspension was placed in a Petri dish with one animal in each Petri dish. The leaf was the only food source for animal. The Petri dishes were kept in a large glass container under controlled conditions in terms of humidity (≥80%), temperature (21±1°C) and light regime (16:8-h light:dark photoperiod). In experiments 1-4, animals were exposed for 3 days, 7 days, 14 and 28 days, respectively. During the 14 and 28 days exposure feces were removed every 7 days to eliminate the possibility of coprophagy. After the exposure the animals were weighed, and anasthetized and decapitated. The digestive glands were isolated and used for assessment of digestive gland cell membrane stability as described below.

### Feeding parameters, weight change and mortality

After 3, 7, 14 or 28 days of exposure of animals to treated leaves, fecal pellets and leaves were removed from the Petri dishes and the leaves were dried at room temperature for 24 h. The dried leaves and fresh animals were weighed and the feeding rate of the isopods was calculated as the mass of consumed leaf per animal’s wet weight per day. The animal’s weight-change in each case was defined as the change in animal wet weight from the beginning to the end of the experiment.

### Digestive gland cell membrane stability

The AO/EB assay is based on the assumption that changes in cell membrane integrity result in differences in permeability of cells to AO and EB dyes. Different permeability to the two dyes results in differentially stained nuclei. Acridine orange is taken up by cells with membranes that are intact or destabilized, and in the cell, emits green fluorescence, as a result of its intercalation into double-stranded nucleic acids. Ethidium bromide on the other hand, is taken up only by cells with destabilized cell membranes, and it emits orange fluorescence, after intercalation into DNA (McGahon et al. 1995). Spectroscopy is used to assess the difference between green and orange emissions, and this provides a measure of cell membrane destabilization.

Cell membrane stability was tested with a modified method described by Valant et al. (2009). After isolation, one hepatopancreatic tube was incubated for 5 min in a mixture of acridine orange and ethidium bromide and then put on a microscope slide. Fresh samples were photographed and examined by an Axioimager.Z1 fluorescent microscope (Zeiss) with two different sets of filters. The excitation filter, 450 to 490 nm and the emission filter, 515 nm (filter set 09) were used to visualize AO and EB stained nuclei, while the excitation filter, 365 nm and the emission filter, 397 nm (filter set 01) were used to visualize nuclei stained with EB alone. Cell membrane integrity was assessed by examination of micrographs. Photographs of intact digestive glands were examined by the same observer twice at intervals of at least 24 h. Cell membrane integrity was assessed visually and classified from 0 to 9 according to a scale of digestive gland cell membrane stability values predefined on the basis of preliminary experiments. This scale defines non-treated (control) animals as showing <5% of nuclei stained by EB, while severely stressed animals have up to 100% of EB-stained nuclei (Valant et al. 2009). In this study the <5% of hepatopancreatic tubes stained with EB were classified as 1 or 2, those with a medium proportion of stained nuclei 3 or 4 and those with the highest proportion (>95%) of EB-stained nuclei as 5 or 6.

### Data analysis

The differences in the medians of measured parameters in exposed and unexposed groups were tested with the non-parametric Mann-Whitney U test. All calculations were done using Statgraphics Plus 4.0 statistics software. Statistical differences between exposed and control animals were categorized into three groups to which different numbers of asterisks were assigned (* *p* < 0.05, ** *p* < 0.01, ***p < 0.001).

## Results

### Characterization of nanoparticles

**Figure 1a, b.**
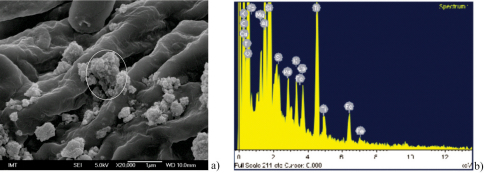
TiO_2_ nanoparticles dispersed over the abaxial leaf surface to give final concentration of 1000 µg/g dry wt of leaf **a** EDX spectrum of area encircled on Figure 1a, where presence of Ti is confirmed **b**.

Scanning electron microscopy revealed the distribution of TiO_2_ particles applied on the lower leaf surface (Fig. 1a) and EDX confirmed their composition (Fig. 1b). The same particles were tested in other studies which provide a more detailed description of their characteristics (Valant et al. 2009). The DLS revealed the hydrodynamic radii of particles in the suspension applied to leaves as 110 nm. The BET method revealed that the surface area of TiO_2_ samples was 144 m^2^/g. The size and surface area correspond to the data provided by the supplier, and the X-ray powder diffraction data confirmed that the TiO_2_ was in the anatase crystal form.

### Feeding parameters, weight change and mortality

The number of exposed animals at the beginning of the exposure and that at the end of the exposure failed to correspond because some animals molted during the course of experiment and consequently were excluded from further analysis. Based on the amount of consumed food it was estimated that when animals were fed on 1000 µg nano-TiO_2_/g of leaf they consumed approximately 0.01 ± 0.01 µg TiO_2_ per day in 3 days, 0.05 ± 0.03 µg TiO_2_ per day in 7 days, 0.07 ± 0.02 µg TiO_2_ per day in 14 days and 0.05 ± 0.02 µg TiO_2_ per day in 28 days. When fed on 2000 µg nano-TiO_2_/g of leaf they consumed approximately 0.08 ± 0.04 µg TiO_2_ per day in 3 days, 0.09 ± 0.03 µg TiO_2_ per day in 7 days, 0.09 ± 0,03 µg TiO_2_ per day in 14 days and 0.1 ± 0.05 µg TiO_2_ per day in 28 days. No significant effect of ingested nano-TiO_2_ on survival and weight change was observed in animals fed with TiO_2_ nanoparticles when compared to control animals fed with untreated food.

**Figure 2.**
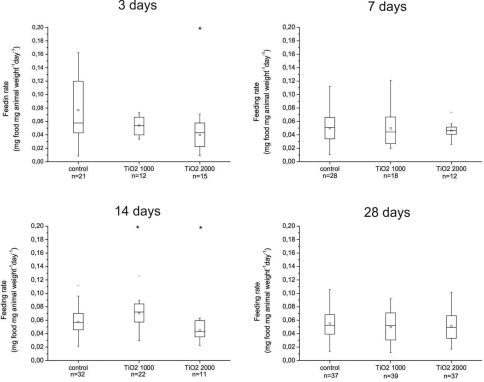
Daily feeding rate (mg of consumed leaves/animal weight) of animals fed on control (untreated) leaves and leaves dosed with 1000 or 2000 µg/g nano-TiO_2_ for 3, 7, 14 or 28 days. On x scale also number of animals in each group is represented (n). There are statistically significant differences between animals exposed to food dosed with 1000 µg/g nano-TiO_2_ for 3 and 14 days compare to control of the corresponding group and between control and 2000 µg/g nano-TiO_2_ in animals exposed for 14 days (* *p* < 0.05). Symbols on the box plot represent minimum and maximum data values (whiskers), mean value (□), 75^th^ percentile (upper edge of box), 25^th^ percentile (lower edge of box), median (line in box) and max and min value ( - ).

There was a statistically significant decrease in feeding rate in animals exposed for 3 days or 14 days on food dosed with 2000 µg/g nano-TiO_2_ when compared with controls (Fig. 2). However, an increase, also statistically significant, occurred in the feeding rate of animals exposed for 14 days to food dosed with 1000 µg/g nano-TiO_2_ when compared with control (p = 0.03). These data indicate a dynamic response of feeding behavior to presence of particles in the food, which was not consistent over time (Fig. 2). In more or less all exposed groups the average feeding rate was similar, indicating reproducibility of the feeding parameters in different experiments.

### Digestive gland cell membrane stability

Our previously published data demonstrate that in animals from a stock culture and in good physiological condition, the digestive gland cell membrane stability classification was higher than 2 in only 5% of animals, and this was considered to be a benchmark (Valant et al. 2009). The higher the value the more the membrane is destabilized.

Our data show that among the control animals fed with uncontaminated food, the digestive cell membranes were affected in up to 10% of exposed animals. We consider this to be a response to suboptimal experimental conditions in terms of isolation of animals, inappropriate shelter during the experiment, and poor food. We consider 10% of animals with affected cell membrane to be normal (benchmark).

**Figure 3.**
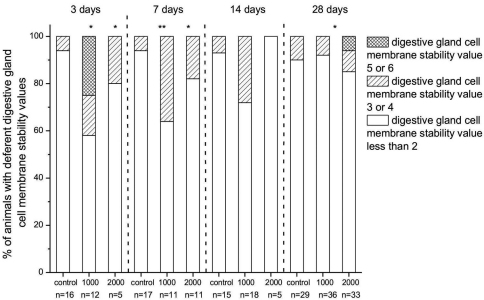
Percentage of animals in fed on food dosed with 1000 or 2000 µg/g nano-TiO_2_ for 3, 7, 14 or 28 days with different degrees of destabilization of cell membranes, assessed visually and classified from 0 to 6 according to the scale defined in Materials and Methods, above. On x scale also number of animals in each group is represented (n). Digestive gland cell membrane stability values ≤ 2 represent animals which had no destabilized cell membrane and digestive gland cell membrane stability values 3 or 4 animals with destabilized cell membranes. Those with value 5 or 6 had the most destabilized cell membranes. Statistical differences between exposed and control animals (within one exposure duration) are marked with an asterisk (* *p* < 0.05 and ** *p* < 0.01).

The most significantly affected groups were those exposed to 1000 µg/g nano-TiO_2_ for 3 days. In this group, 25% of animals had a cell membrane destabilization value of 5 or greater. After 7 and 14 days of exposure to food dosed with 1000 µg/g nano-TiO_2_, digestive cell membrane destabilization was detected in 36% and 28%, respectively, of the exposed animals, but after 28 days of feeding on food dosed with 1000 µg/g nano-TiO_2_ there was almost no effect (8%) on digestive gland cell stability. The highest concentration of TiO_2_ particles in food (food dosed with 2000 µg/g nano-TiO_2_) investigated was generally less harmful to digestive cell membranes, although in some (6%) of the animals exposed for 28 days, serious destabilization of the digestive cell membranes was observed (Fig. 3). These severe biological effects (after 3 and 28 days of exposure) were neither dose- nor duration-related and this cell membrane damage, which was never seen in control animals, could be interpreted as a sporadic effect.

## Discussion

In our study no toxic effects could be confirmed by conventional toxicity parameters such as weight change or mortality in short-term (3 and 7 days) and prolonged (14 and 28 days) exposure. The changed feeding rate of exposed animals compared to controls is also convenient evidence of the effects of chemicals on isopods. We hypothesize that the adverse effect of chemicals is manifested in a reduced feeding rate. In cases where the feeding rate significantly increases, it is thought to be a hormetic like response (Drobne et al. 2008). Hormetic like response means that exposure to substances stimulate and not retard the measured response. This is a case when organisms are exposed to lower concentrations of substances and when effects at higher levels of biological complexity are recorded.

In this study, the feeding rate of the animals increased, decreased or was not affected at all. These observations coincide with our previous results in which nanosized TiO_2_ enhanced the feeding rate of *Porcellio scaber* (Drobne et al. 2008) while some other chemicals caused reduced feeding activity (Drobne and Hopkin 1995).

Different studies report changed feeding rates after feeding animals on chemically dosed food for different periods of time. For example, the feeding rate in *Porcellio scaber* was assessed after 3 days (Drobne et al. 2008), 14 days (Drobne et al. 2008, Stanek et al. 2006), 21 days (Staak et al. 1998, Kolar et al. 2010), 28 days (Zidar et al. 2009, Zizek et al. 2011) and 35 days (Drobne and Hopkin 1995) of exposure to different substances. In juveniles, the feeding rate was statistically significantly reduced when animals were fed for 3 days with 50 µg/g of the pesticide imidacloprid, while in adults it was reduced when 10 µg/g of imidacloprid were incorporated in the diet (Drobne et al. 2008). No effect was found, however, on the feeding rate in *Porcellio scaber* after exposure for 14 days to the pesticide diazinon (Stanek et al. 2006) at levels up to 100 μg/g. Staak et al. (1998) failed to observe any response after 21 days of exposure to the herbicide trifluralin. Kolar et al. (2010) reported that the antiparasitic veterinary drug abamectin (NOEC = 3 mg/kg dry soil) significantly reduced the food consumption rate in *Porcellio scaber* at levels of 10 mg/kg in dry soil after 21 days exposure, but no effect was observed after 28 days exposure of isopods to the polyether antibiotic monensin (NOEC ≥ 849 mg/kg dry soil, NOEC ≥ 357 mg/kg dry food) (Zizek et al. 2011). There are also reports of studies on the effects of metals (Zn, Cu, Co and Cd) on *Porcellio scaber*. Zidar et al. (2009) reported that after 28 days of exposure to metals, a dose-related decrease in food consumption rate was observed, when a mixture of Zn and Cd was included in the food at nominal levels (2600 mg Zn + 360 mg Cd/kg dry food). The same group (Zidar et al. 2003) also documented a reduced feeding rate after exposure of the animals to 1800 μg Zn/g, 1200 μg Cu/g or 125 μg Cd/g food for two weeks. Drobne and Hopkin (1995) observed a reduction of food consumption after 35 days exposure of terrestrial isopods to 2000 μg Zn/g in their food. In another study, Drobne and Hopkin (1994) reported a reduced feeding rate as a result of feeding on Co-dosed food. In this case, 500 μg/g Co in food led to a slight, statistically insignificant effect on the feeding rate, while 2500 μg/g Co in the food significantly reduced the feeding rate after 3 weeks of exposure. Knigge et al. (2000) reported that after 80 days of exposure the applied lead concentrations at a maximum of 7945 mg/kg food dry weight had no significant quantitative effect on food consumption by isopods, although a population pre-exposed in an artillery range showed a tendency towards food uptake higher than that of the control population. Jereb et al. (2003) reported a reduction in the feeding rate after exposure to 300 μg of the radiotracer ^203^Hg^2+^/g leaf for 7 days but no difference in the food consumption was observed in animals that were exposed to 0.3 µg ^203^Hg^2+^/g leaf for 16 days and to 3.0 µg ^203^Hg^2+^/g of leaf for 16 or 35 days.

Feeding rate changes appear to be a suitable measure of effects of ingested chemicals. Whether this is a convenient measure of effects of nanoparticles is needed to be confirmed in future research. Data obtained with nanoparticles suggested that feeding rate changes are neither dose nor time dependent. Feeding rates of exposed animals either increased or decreased when compared to controls. Such result may indicate that: (a) exposure duration was not long enough to provoke effect; (b) exposure concentration was too low to exert effect or (c) nanoparticles have stochastic type of effects which occur by chance and are not time nor dose dependant. To confirm this is a change for future research.

In the study presented here feeding rates were not severely affected even at exposures of up to 28 days, but an effect was seen at shorter exposure duration. Consequently, a concentration of nano-TiO_2_ of 2000 µg/g in the food may not be assumed to be a “no observed effect concentration” (NOEC).

In contrast to the not so significant effect on standard toxicity parameters in our study the cell membranes of digestive glands in almost half of exposed animals (42%) fed on 1000 µg/g nano-TiO_2_ were destabilized after as little as 3 days of exposure. After 7 days of exposure to food containing 1000 µg/g nano-TiO_2_, digestive cell membrane destabilization was detected in 36% of the exposed animals. Animals exposed for longer periods, 14 or 28 days, did not exhibit such intensive membrane damages as was expected, but in animals exposed for 28 days to highest exposure concentration, the digestive gland membrane was severely damaged, a result that was never observed in controls. We conclude that the severe damage of membrane was neither dose- nor exposure duration dependent but occurs sporadically. Here again, we observed different type of response to nanoparticles when compared to non-nanoparticulate chemicals. Performed with soluble chemicals, the AO/EB assay reveals a dose response effect (Valant et al. 2009).

A moderate effect was found to be more common in animals exposed to lower concentrations and for shorter times. In the light of currently available knowledge we speculate that in such cases, the cell membrane is destabilized, but the organism has a mechanism to restore its normal activity. The ability of cells to alter their lipid composition and thus their rigidity after exposure to CuO nanoparticles has been demonstrated by Mortimer et al. (2011).

We speculate that in our study, TiO_2_ nanoparticles interact first with the cell membrane, and this interaction is diagnosed as cell membrane destabilization. Subsequently, the cells respond to this destabilization of the cell membrane by repairing its stability. This is indicated by the failure to observe intensification of cell membrane destabilization after prolonged exposure durations, such as 28 days. Not with standing this, the cell membrane in some animals was severely affected. Future research is needed to learn if this severe damage could lead to toxic effects or if it can be reversed.

## Conclusions

We found nano-TiO_2_ to manifest no severe toxicity after 3, 7, 14 or 28 days of dietary exposure to 1000 µg/g or 2000 µg/g of nano-TiO_2_when measured by conventional toxicity measures such as feeding rate, weight change, and mortality.

Severe cell membrane destabilization was sporadic, and was independent of dose and duration of exposure.

The highest tested concentration with 28 days of exposure is not the NOEC because the membrane destabilization effects were observed at shorter duration periods.

The toxic effect of nanoparticles has to be interpreted differently from that of soluble chemicals. It appears more a stochastic-like effect which is not dose responsive.
